# Use of Audiobooks as an Environmental Distractor to Decrease State Anxiety in Children Waiting in the Pediatric Emergency Department: A Pilot and Feasibility Study

**DOI:** 10.3389/fped.2020.556805

**Published:** 2021-01-05

**Authors:** Leah I. Stein Duker, Anita R. Schmidt, Phung K. Pham, Sofronia M. Ringold, Alan L. Nager

**Affiliations:** ^1^Division of Occupational Science and Occupational Therapy, University of Southern California, Los Angeles, CA, United States; ^2^Division of Emergency and Transport Medicine, Department of Pediatrics, Children's Hospital Los Angeles, Los Angeles, CA, United States; ^3^Keck School of Medicine at the University of Southern California, Los Angeles, CA, United States

**Keywords:** distraction, environment, pediatrics, audiobook, fear, state anxiety, emergency department (ED)

## Abstract

**Objectives:** Anxiety and anticipatory stressors are commonly experienced by children visiting the Pediatric Emergency Department (PED), but little research exists that addresses the efficacy of interventions to decrease this stress. This one-sample pretest-postest pilot study gathered preliminary data on the feasibility and effectiveness of utilizing audiobooks to reduce fear and state anxiety in children in the PED.

**Methods:** Participants were 131 children in kindergarten through 8th grade (*M* = 9.4 years, 54% female), triaged urgent or emergent, presenting to the PED. Participants self-reported fear (Children's Fear Scale) and state anxiety (modified State-Trait Anxiety Inventory for Children; mSTAIC) before and after listening to an age-appropriate audiobook (two options). Data regarding patient experience were also collected. Paired samples *t*-test was used to examine pre–post intervention changes in fear and state anxiety.

**Results:** Significant, albeit small, improvements in fear and the mSTAIC states of nervous, calm, happy, and relaxed were found after use of the audiobook (Cohen's *d*_*z*_ = 0.22–0.35). Small, yet significant correlations were found between child age/grade level and improvements in fear and in the mSTAIC states of scared and relaxed, suggesting that the audiobook was more beneficial for older participants. Over 60% of participants liked the audiobook content “a lot” as well as enjoyed listening to the audiobook “a lot.” Without prompting, 15% of participants requested to listen to an additional audiobook.

**Conclusions:** Listening to an audiobook is feasible and could be effective in decreasing fear and state anxiety for children during a waiting period in the PED. The technology is low-cost, simple, and portable. The results of this study should be interpreted with prudence due to the lack of a control group and results that, although significant, were modest based on effect size conventions; future studies should explore the impact of audiobooks on patient stress with an expanded sample size and control group.

## Introduction

Feelings of anxiety, stress, and fear before healthcare procedures can have psychological and physiological effects, adversely interfering with optimal patient outcomes (1). For example, anxiety and anticipatory stressors are commonly experienced by children during visits to the Emergency Department, which may engender uncooperative behaviors and difficulty tolerating medical procedures. Emotional and behavioral challenges following treatment may have the potential to negatively impact physical recovery (2). Research in the ED has focused primarily on pharmacological and non-pharmacological methods to minimize procedural pain, often during venipuncture or laceration repairs (3–11).

However, research suggests that between 36 and 50% of children may experience heightened state anxiety simply from being present in the Pediatric ED (PED), without even undergoing a procedure (2, 12). Minimal research has investigated the efficacy of interventions to decrease anticipatory anxiety, stress, and fear in the PED. Thus far, research has examined the use of ambient lighting, music, and aromatherapy in the waiting room, as well as the utilization of Child Life and hospital clowning with varying success (12–14).

Since situational anxiety and anticipatory stressors have the potential to lead to negative outcomes, it is essential to identify feasible and efficacious interventions to decrease anxiety and fear in children in the PED. Audiobooks are a low cost and easily accessible distraction technique, which are growing in popularity. Therefore, the purpose of this pilot study was to gather preliminary data on the feasibility and effectiveness of utilizing audiobooks to reduce fear and state anxiety in children in kindergarten through 8th grade in the PED during a waiting gap in care.

## Methods

This was a prospective, one-sample pre-post-test study that took place at a University-affiliated children's hospital located in a large metropolitan area of the United States, which sees almost 95,000 pediatric patient encounters per year. This study was approved for human subjects by The Institutional Review Boards of the children's hospital and the affiliated University.

### Participants

**Figure 1:**
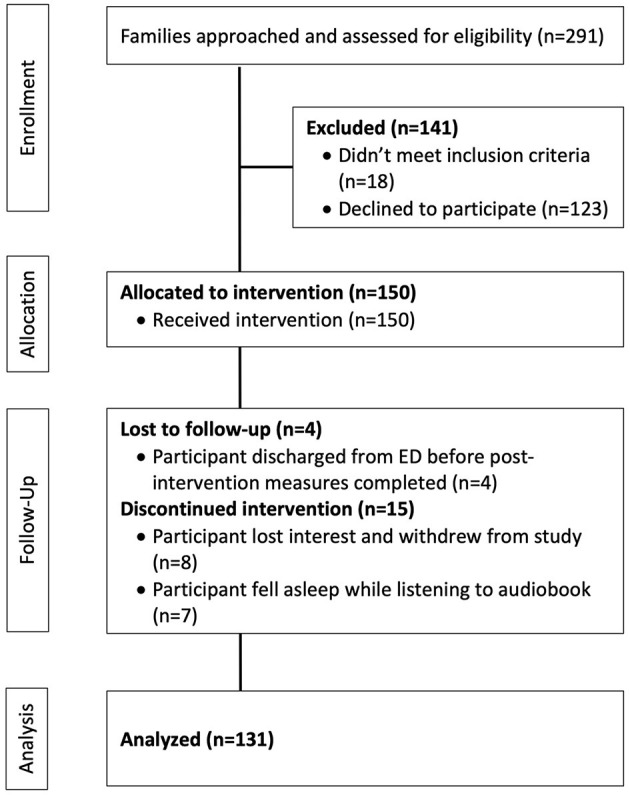
Study flow chart.

Participants included a convenience sample of 150 children presenting to the PED between April 2018 and May 2019 (see Figure 1). As the primary purpose of this study was to assess the feasibility and preliminary effectiveness of our intervention, we surmised a priori that a convenience sample of at least 100 participants would generate enough data to achieve our primary aim (15, 16).

Eligibility criteria included English-speaking children in kindergarten through 8th grade, triaged emergent or urgent [Emergency Severity Index (ESI) levels 2 and 3], with an English-speaking caregiver or parent. Exclusion criteria included children with hearing difficulties that would impact the audiobook experience, and/or a development delay that would prevent the child from answering questions or understanding the story. ESI level 4 and 5 (semi-urgent and non-urgent) children were excluded due to supposition that these lowest acuity PED visits may be too short in duration to complete all study activities; ESI level 1 (critical) children were excluded due to concerns that the intervention may interfere with the life-saving care they required.

### Procedures

Study team members, including non-clinical research staff and trained health science undergraduate research assistants, monitored the PED electronic tracking board for eligible children. Following physician permission and family interest, child assent and parent permission procedures were completed in participants' respective examination rooms. Each child participant then answered the Children's Fear Scale [CFS (17)] and our modified version of the State-Trait Anxiety Inventory for Children [STAIC (18)], described subsequently in greater detail. Immediately after, the child participant chose one audiobook from a list of two age-appropriate options, and listened to one story utilizing disposable ear buds. Stories were chosen from a Penguin Random House audio repository of their titles by a hospital-based literacy specialist. All stories were presented in English, on an iPad affixed to a mobile stand with no illustrations in each participants' examination room. Once an audiobook was started, research team members left the room and children were not able to speed, scrub, or change the story location; stories could only be paused during a medically-related interruption. While the child participant listened to the audiobook, a parent completed child descriptor questions (e.g., race and ethnicity, current/previous mental health diagnoses, and current use of medications for mental health diagnoses). Following the completion of the audiobook, children again answered the CFS and modified STAIC as well as three questions probing their experience. Additional data were collected from the electronic medical record by the study team at the culmination of study participation (e.g., age, sex, length of stay, and final diagnosis).

### Outcomes

#### Fear

The CFS (17) was utilized to assess child fear before and after listening to the audiobook. This child-report scale consists of drawings of five faces that express increasing fear, with the child asked to point to the face that best fits his or her level of fear varying from “*not scared at all*” *to* “*the most scared possible*.” The CFS is scored from 0 to 4, with the higher score indicating greater fear (17). This assessment has documented high reliability and validity (17) and has been used to examine fear and anxiety during activities such as venipuncture (17), intramuscular injection (19), and blood draws (20).

#### State Anxiety

The STAIC (18) is a commonly utilized tool to assess participant anxiety in research. However, it is validated only for children 9–12 years, so studies with younger populations, such as ours, must choose another tool (9, 21). Additional critiques exist, focusing on challenges with item comprehension as well as the length of time required for completion in clinical settings (22–24). Research also suggests that certain items are commonly left unanswered when the STAIC is completed in medical settings (25). This is problematic as more than two item omissions renders the STAIC score as invalid (18), with the mean age of children omitting more than two items 8.0 years (25), which is included in our target study population.

Therefore, our study team of individuals with expertise in anxiety, distraction interventions, emergency medicine, child development, research instrument development, and hospital-based literacy, met iteratively and through multiple sessions to develop a modified STAIC for use in this study. In doing so, we utilized data regarding frequency of item omission (25) and took into account the previously developed “short STAI” for adults (26) which has inconsistent psychometrics when used for children (22, 27). The resulting tool contained six items, comprised of three positive words (calm, relaxed, and happy) and three negative words (nervous, upset, and scared) from the original STAIC. For each item, the child made a selection that described how he/she felt at that very moment. For example, *I feel: very calm, calm, not calm*; *very* items were scored as 1, stem word alone as 2, and *not* as 3. The six items were analyzed separately rather than as a summed total.

#### Patient Experience

Participants were asked three questions to ascertain their experience of the audiobook intervention. These included: (1) how much they liked the audiobook content, (2) how much they enjoyed listening to the audiobook, and (3) the degree to which they were bothered by medical interruptions while listening to the audiobook. All questions were scored on a 1 (not at all) to 4 (a lot) Likert scale; the degree of bother from interruptions question also had a not applicable/no interruptions answer choice.

### Data Analysis

In order to examine the effectiveness of the full exposure of the audiobook intervention, discontinuations (e.g., lost interest, fell asleep) and/or withdrawn participants were excluded from analyses (see Figure 1). Data were analyzed using SPSS software version 23 (IBM Corp., Armonk, NY USA). Summary statistics (e.g., frequencies, means, and standard deviations) were used to examine sample characteristics and participant experience, and chi-square tests examined whether the patients who enrolled differed from those who declined, based on PED triage level and school grade. Paired samples *t*-test was used to examine pre–post intervention changes in fear and state anxiety (results were checked using the non-parametric Wilcoxon test). Pre–post differences in fear and state anxiety were computed for each individual participant and examined in the following ancillary analyses: Pearson's correlation test with child age and school grade level (results were checked using the non-parametric Spearman's correlation test); one-way analysis of variance models with mental health status (yes or no), ED triage level (urgent or emergent), and final diagnosis (six categories) as separate between-subjects variables (results were checked using the non-parametric Kruskal–Wallis test). All analyses were conservatively two-tailed and conducted at the 0.05 significance level.

All 22 items of the Strengthening the Reporting of Observational Studies in Epidemiology (STROBE) reporting guidelines were addressed (28).

## Results

### Study Sample

Between April 2018 and May 2019, 55% of eligible children were enrolled (*n* = 150; see Figure 1). Participant mean age was 9.4 years, 54% were female; and 63% were Hispanic/Latinx (see Table 1). The most common chief medical complaints were pain and/or trauma (29%) or a systemic complaint (e.g., testicular complaint, eye complaint, swelling/lesion; 28%). Prior to the intervention, participants reported that they were feeling a little scared (*M* = 0.85, SD = 1.09). Participants also reported that they were minimally scared, upset, and nervous, while also moderately calm, happy, and relaxed (see Table 2). Patients who declined participation were not significantly different from those who accepted based on ED triage level [*X*^2^(1) = 1.30, *p* = 0.25] and school grade level [*X*^2^(8) = 7.76, *p* = 0.46].

**Table 1:** Descriptive characteristics of participating children.

	**Participants (*n* = 131)**
	**Mean (SD)**
Age	9.4 (2.3)
Length of Stay (hours)	2.7 (3.4)
	***N*** **(%)**
**Sex**	
Female	71 (54)
Male	60 (46)
**Race and Ethnicity**	
Hispanic/Latinx	83 (63)
Black/African American	13 (10)
White	11 (8)
Asian/Pacific Islander	5 (4)
Other/Mixed	16 (12)
Not Reported	3 (2)
**Final Diagnosis**	
Pain and/or trauma	38 (29)
Systemic Complaint	36 (28)
Gastrointestinal	24 (18)
Respiratory	24 (18)
Simple Infection	6 (5)
Other	3 (2)
**Mental Health Status (e.g., depression, anxiety, and ADD/ADHD)**	
No Mental Health Diagnosis	104 (79)
Mental Health Diagnosis	26 (20)
No data reported	1 (1)

**Table 2:** Fear and state anxiety levels before and after the audiobook intervention.

**Variable**	***N***	**Mean**	**SD**	**Pre-post**			***t*(df)**	***p***	**95% CI of mean difference**	**Cohen's *d_***z***_***
				**Mean difference**	**SD**	**SE**				
Pre-fear	131	0.85	1.09	0.34	1.06	0.09	3.61 (130)	<0.001	0.15 to 0.52	0.32
Post-fear	131	0.51	0.95							
Pre-scared	131	2.65	0.54	−0.08	0.61	0.05	−1.58 (130)	0.12	−0.19 to 0.02	0.14
Post-scared	131	2.73	0.57							
Pre-upset	131	2.85	0.38	−0.02	0.40	0.04	−0.65 (130)	0.52	−0.09 to 0.05	0.06
Post-upset	131	2.87	0.42							
Pre-nervous	131	2.51	0.67	−0.20	0.66	0.06	−3.44 (130)	0.001	−0.31 to −0.08	0.30
Post-nervous	131	2.71	0.55							
Pre-calm	131	1.84	0.67	0.28	0.79	0.07	4.00 (130)	<0.001	0.14 to 0.41	0.35
Post-calm	131	1.56	0.68							
Pre-happy	128	1.90	0.66	0.19	0.67	0.06	3.15 (127)	0.002	0.07 to 0.31	0.28
Post-happy	131	1.71	0.69							
Pre-relaxed	131	1.85	0.70	0.17	0.76	0.07	2.54 (130)	0.01	0.04 to 0.30	0.22
Post-relaxed	131	1.68	0.65							

*Wilcoxon test (non-parametric) results showed patterns consistent with the above paired samples t-test (parametric) results*.

### Preliminary Effectiveness

#### Main Results

Mean fear and state anxiety levels before and after the audiobook intervention are shown in Table 2. Pre–post mean differences in fear and in the states of nervous, calm, happy, and relaxed were statistically significant. On average, participants felt less fearful and nervous as well as more relaxed, calm, and happy after listening to an audiobook. These effect sizes were modest, based on effect size conventions (29–31).

#### Ancillary Results

Child age and school grade level were significantly correlated with pre–post differences in fear [*r*_*s*_ = 0.18 (age and grade), *p*'s = 0.04] and in the modified STAIC states of *scared* [*r* = −0.21 (age), *p* = 0.02; *r* = −0.19 (grade), *p* = 0.03] and *relaxed* [*r* = 0.18 (age), *p* = 0.05; *r* = 0.18 (grade), *p* = 0.04]. These results suggest that the audiobook was more beneficial for older participants; with greater age, participants experienced a larger reduction in fear and feeling scared alongside a greater increase in feelings of relaxation. Mental health status, ED triage level, and final diagnosis were all non-significant as between-subjects variables, indicating that pre–post differences in fear and state anxiety were not differentially affected by any of these factors.

#### Patient Experience

Although 62% (*n* = 80) of participants experienced medical interruptions, most were minimally bothered by these interruptions; 37 (46%) were not at all bothered and 29 (36%) were only a little bothered. The majority of participants (69%) liked the audiobook content *a lot* as well as enjoyed listening to the audiobook *a lot* (63%). Without prompting, 19 (15%) of participants requested to listen to additional audiobooks upon completing the study activities.

## Discussion

This study aimed to explore both the feasibility and preliminary effectiveness of an audiobook intervention to reduce fear and state anxiety in children in kindergarten through 8th grade in the PED. While our results coalesced with much of the existing literature on state anxiety in the PED, several exceptions can be seen. First, our study sample reported only minimal state anxiety and fear, with a majority labeling themselves as only “a little scared”; both Nager et al. (2) and Heilbrunn et al. (12) identified PED patients as being significantly more fearful and anxious. Though the sample ages in our study and the one by Heilbrunn et al. (12) were similar, Nager et al. (2) studied a noticeably older sample (mean 13.3 years); as such it is possible that the differences in observed state anxiety could be attributed to the increased situational awareness that typically accompanies adolescence. Moreover, a significant number of families declined to participate in our study, which is not unusual for a prospective clinical research study in the PED. Though the patients who declined did not differ from the study sample in terms of triage level and grade, it is possible that less anxious families self-selected for participation. Alternatively, all child participants were aware that an audiobook would be provided to them as part of their study participation, with the assent stating that “We want to see if listening to an audiobook can make this time easier for children like you.” It is possible that the language used in recruitment and assent procedures led to a priming effect, with children experiencing some anticipatory stress relief upon knowing they would be provided an audiobook to improve their experience in the PED.

The instruments we selected to assess our primary outcomes shared some overlap and several differences between those used in previous studies. While Nager et al. (2) used the STAIC and the anticipatory stressors checklist (a tool assessing the presence of 10 current fears or worries), Heilbrunn et al. (12) chose the modified Yale Preoperative Anxiety Scale (32) (m-YPAS; a 5-item observational tool to assess anxiety at different times during a medical encounter). Since research examining the use of these instruments concurrently in the PED has not yet been done, it is difficult to state whether the differences in measured state anxiety were due to the underlying populations or the sensitivity of the instruments themselves. This gap in knowledge underscores the need for psychometrically sound tools that are both developmentally appropriate and validated for the PED setting. Additionally, although it is hard to determine what numerical value corresponds to a clinically meaningful reduction in stress, it could be argued that any relief can be interpreted as being advantageous to the patient.

Interestingly, our results found a significant improvement on the CFS measure but not on the mSTAIC item “scared.” Research suggests that use of faces scales may be advantageous to obtain pediatric self-report measures (17, 33–35). Although the CFS has been shown to exhibit moderate-high validity with other self-reports of fear (17), it is possible that the CFS was more sensitive in capturing data regarding fear experience from the children in our study, as compared to the mSTAIC, explaining the significant improvement in fear on the CFS, but not on the “scared” item of the mSTAIC. Alternatively, the mSTAIC includes a more restricted range of responses as compared to the CFS; however, simple Likert scales with descriptor words as anchors are commonly used and considered appropriate for children (36). More research is needed to identify the mechanisms which may underlie this discrepancy.

### Feasibility

Audiobooks are a feasible and low-cost intervention for the reduction of state anxiety in the PED. There was minimal burden of running this intervention; the ability to move the iPads easily from room to room and to clean them between patient uses makes them well-suited to our unique context. In addition, children were willing to use the single-use ear buds we provided and there were no problems with the technology needed to access the audiobooks. Use of an audiobook distraction could easily be added to existing hospital-based stress reduction toolkits (e.g., Child Life). Despite the fact that a majority of children experienced medical interruptions during the audiobook listening period, most reported being minimally bothered, which further supports the use of an audiobook intervention in the often chaotic PED. Lastly, all children were willing and able to respond to our battery of pre- and post-test questionnaires.

Audiobooks can be played on different types of equipment (e.g., Amazon Fire Tablet, iPad, and smartphones) which range in cost from ~$50–$150 (Amazon Fire; previously known as Kindle Fire tablets) or starting at ~$300 (iPad). Audiobooks themselves also range in price, with cost often tied to the length of the recording. For example, a children's book lasting only a few minutes may cost $5.00 while a middle-grade chapter book with a length of 3–4 h may cost $15.00. However, many audiobooks can be found for free online from local libraries or other sources. Single-use earbuds can be purchased for as low as $0.50 per unit. In sum, the costs of purchasing the equipment and books for an audiobook intervention in a hospital is minimal. It is also important to consider that many patients will come to the PED already with the necessary equipment to utilize a hospital-provided audiobook intervention (e.g., smartphone, tablet, iPad, and/or headphones).

More than half of families approached were interested in using our audiobooks intervention, suggesting the desirability of this type of intervention, despite families' access to smartphones, TV, and Child Life services in the PED. While distraction kits for children in the ED have been previously described (37), hospitals may consider integrating audiobooks into the choice of available strategies and working with Child Life or hospital-based literacy specialists to ensure that the offerings are developmentally appropriate. This medium could additionally offer children the opportunity to become familiar with the PED and specific procedures through the use of hospital-based stories, and the use of audiobooks could feasibly extend to any other area of the hospital.

### Future Research

Future research should consider the development of a state anxiety measure specific to the PED. This would allow for coherent interpretation of anxiety scores across research studies with attention to context. Since parental anxiety has the potential to influence a child's anxiety, pain, and cooperation, investigators should also consider exploring parental anxiety as a covariate for the effectiveness of anxiety-reducing interventions in the PED (38). Also, with the advent of new technologies that facilitate non-laboratory based collection of physiological data, future research should integrate objective physiological measurements that reflect stress and anxiety (e.g., blood pressure, heart rate, and electrodermal activity) into future ED stress-reduction interventions in addition to self-report measures.

Comparing audiobooks to other distraction-based interventions (e.g., music, videos, games, bubbles, kaleidoscopes, and guided imagery) may also be of interest in forthcoming research, as some of these interventions have reported to be effective in reducing anxiety and pain in children in medical settings, especially when matched to the developmental level of the child (39–42). While this study explored the audiobook intervention for children in kindergarten through 8th grade, it is worth noting that the transition from picture books to chapter books typically occurs between grades 2 and 3, depending on the child (43)^1^, (44)^2^ Future researchers should explore the use of an audiobook intervention in the PED that incorporates illustrations for children grades 3 and under.

As personal characteristics such as pain, presence of a chronic condition, and socioeconomic factors may influence the presence and experience of stress and anxiety (38, 42, 45), future research should also take into account these factors in study design and analyses. Although pain was not assessed in the current study, ED triage level did not significantly impact fear and state anxiety in the current analyses. Likewise, we did not collect information about participant chronic conditions; however, this study focused on the impact of audiobooks to diminish anxiety with the acute nature of their PED visit, regardless of participants' previous hospital experiences or chronic conditions. Lastly, although it can be difficult recruiting diverse populations in research (46), 73% of our sample self-reported as Hispanic/Latinx or Black/African American. Although our study implemented the intervention during a waiting gap in care in patient examination rooms, it should be feasible to utilize audiobooks during waiting gaps in care in other locations, such as the PED waiting room. Determination of effectiveness of audiobooks to diminish anxiety and fear in these other locations should be examined in future research.

### Limitations

This study had several limitations. First, the study was based on a convenience sample of English-speaking patients and caregivers due to lack of access to Spanish language audiobooks and research assistant availability. As a result, a large portion of the hospital's predominantly Hispanic/Latinx population was excluded, although 63% of our sample did self-report as Hispanic/Latinx. Next, our sample may have represented a less stressed cohort than previous studies. However, even with low levels of pre-intervention stress and anxiety, use of the audiobook was able to significantly improve scores on the CFS and four of the six modified STAIC items. Future research should re-examine the use of audiobooks to decrease anxiety with a control group in order to examine if this decrease in anxiety was due to the intervention or acclimation to the PED. Our modified STAIC instrument was *de novo*, and therefore only had face validity, but we argue that it enabled ecological validity of anxiety assessment within the PED context. Through our modifications to the STAIC, we aimed to shorten completion time by reducing survey items as well as increasing patient understanding of anxiety state descriptor words. While the length of the audiobooks within age categories were similar, the length of the books between age categories were different. As a result, it is possible that study results were confounded by book length.

## Conclusion

Preliminary evidence suggests the use of audiobooks could be effective to improve children's experience of fear and state anxiety in the PED, despite the presence of medically-related interruptions. Audiobooks are low cost, easy to implement, and a portable intervention that responds to the American Academy of Pediatrics' endorsement of a “relaxing environment [to] help a child to feel more comfortable and less stressed” in the PED [(38), p. e1393]. However, it will be important replicate these findings due to multiple limitations, including the absence of a control group and results that, although significant, were modest based on effect size conventions.

## Data Availability Statement

The raw data supporting the conclusions of this article will be made available by the authors, without undue reservation.

## Ethics Statement

The studies involving human participants were reviewed and approved by Children's Hospital Los Angeles Institutional Review Board and the Health Sciences Institutional Review Board of the University of Southern California. Written informed consent to participate in this study was provided by the participants' legal guardian/next of kin; additionally, participant assent was obtained as appropriate.

## Author Contributions

LSD, AN, AS, PP, and SR designed the study. AS and SR acquired data. PP analyzed the data. LSD wrote the first draft of the manuscript. All authors contributed to the article and approved the submitted version.

## Conflict of Interest

Penguin Random House donated the audiobooks and iPads for this study but was not involved in the study design, collection, analysis, data interpretation, or dissemination of study results.

## Footnotes

^1^https://www.scholastic.com/parents/books-and-reading/reading-resources/book-selection-tips/world-kids-chapter-books.html

^2^https://www.romper.com/p/what-age-should-kids-read-chapter-books-heres-how-to-know-its-time-for-the-big-books-8885872.
