# Supplementary material for: Use of Audiobooks as an Environmental Distractor to Decrease State Anxiety in Children Waiting in the Pediatric Emergency Department: A Pilot and Feasibility Study
Source: Front Pediatr. 2021 Jan 5;8:556805. doi: 10.3389/fped.2020.556805 (PMC7874121; doi:10.3389/fped.2020.556805)
Supplement: Supplementary file 1 [file Data_Sheet_1.PDF]

Supplementary Material

1 Supplementary Table

Supplementary Table 1. Audiobook descriptions by age range

Age Range	Story Choice	Description	Story Duration
Kindergarten – Grade 2	Nate the Great Collected Stories	The World's Greatest Detective *Nate the Great* helps his friend Annie find a lost picture. As he solves the case, he also finds Super Hex, the lost cat!	12:31
	The Story of Ferdinand	All the other bulls like to run, jump and butt their heads together. *Ferdinand* would rather sit and just smell the flowers. But one day, a bumblebee changes his life!	9:42
Grades 3 – 5	Sideways Stories from Wayside School	Meet the kids of Wayside School: Todd, who just can't seem to behave; Bebe, who draws faster than anyone; and Kalvin, who accomplishes an impossible task!	16:11
	Jake the Fake	Jake can barely play an instrument or draw a stick figure, which is a real problem because he just faked his way into the Music and Art Academy for the Gifted and Talented!	20:45
Grades 6 – 8	Secret Samantha (Flying Lessons)	Samantha isn't one of the most popular kids in school, and now she has to pick a Secret Sharers present for the cool new girl in her class.	18:51
	Fantasy League	Charlie is a twelve year old fantasy football pro. Although he's just a bench warmer, he knows more about the game than anyone else on the field.	16:19

2 Supplementary Figures

Supplementary Figure 1. Audiobook set-up.

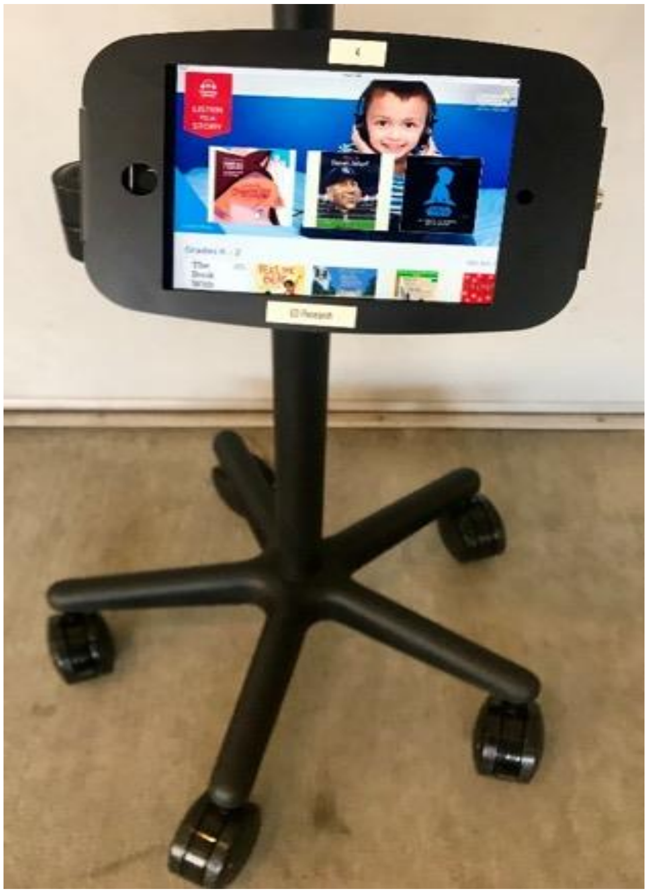

Supplementary Figure 2. Mean child modified STAIC (positive state) scores before and after audiobook intervention.

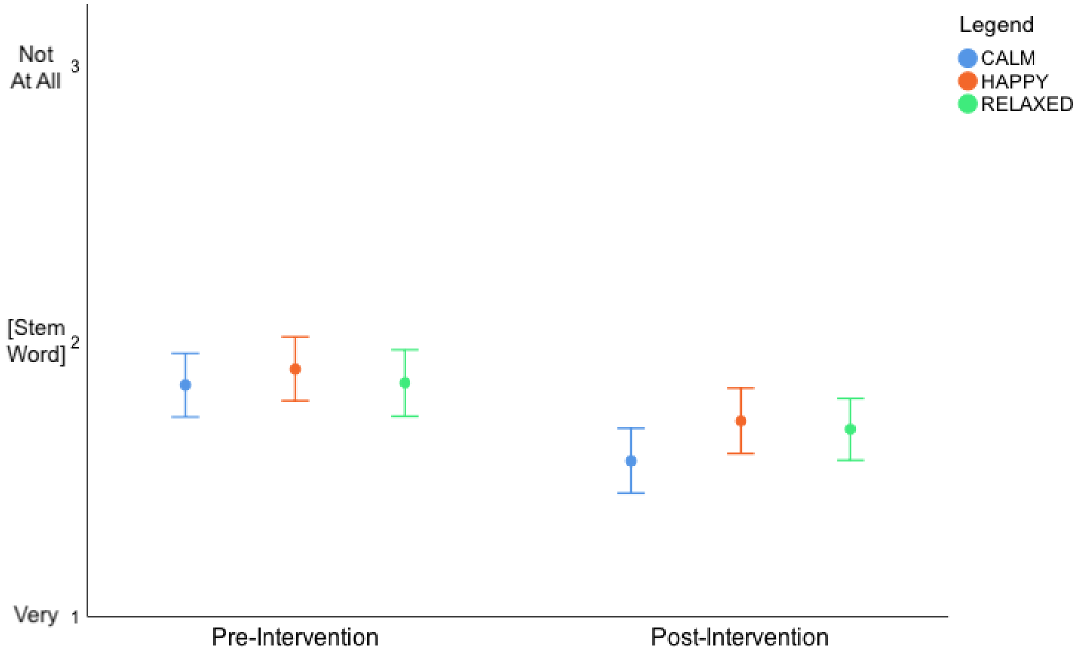

Supplementary Figure 3. Mean child modified STAIC (negative state) scores before and after audiobook intervention.

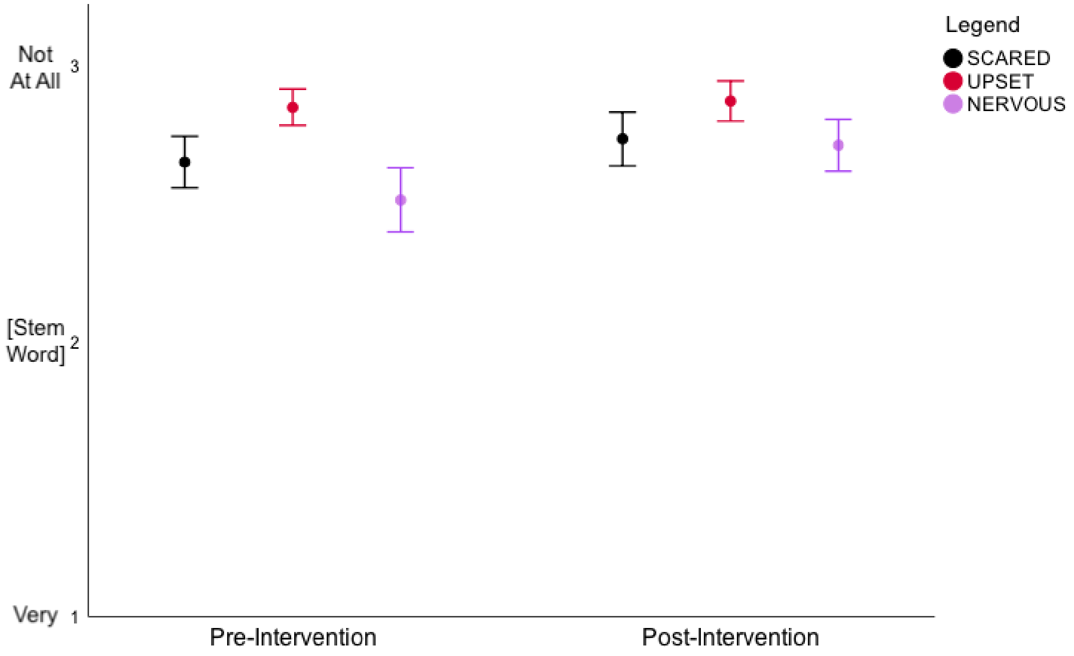

Supplementary Figure 4. Mean child fear scores before and after audiobook intervention.

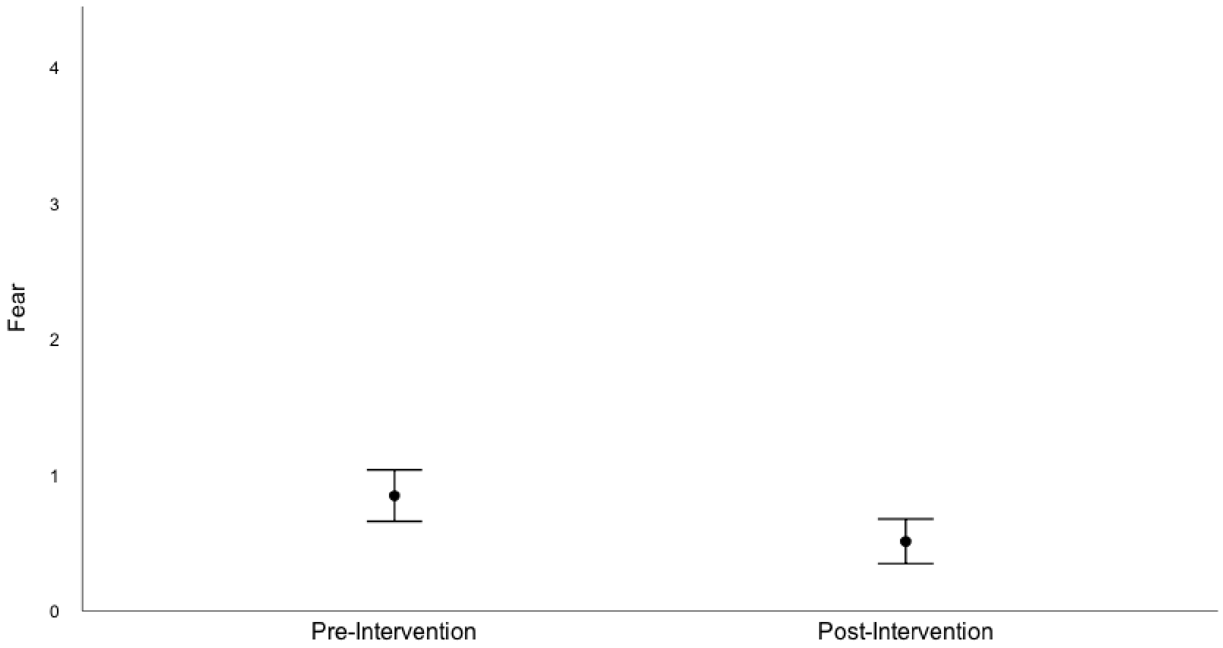

3 Supplementary Appendix

Supplementary Appendix 1. Strengthening the Reporting of Observational Studies in Epidemiology (STROBE) Checklist.

No. Topic	Item	*Response*
**Title and abstract**		
1 Title	Indicate the study's design with a commonly used term in the title or the abstract.	*We have modified the abstract to indicate the research was a one-sample pretest-posttest study design.*
1 Abstract	Provide in the abstract an informative and balanced summary of what was done and what was found.	*Our abstract provides a balanced summary of what was done and found; we have additionally included quantitative findings (p-values) in the abstract to provide greater information.*
**Introduction**		
2 Background/ Rationale	Explain the scientific background and rationale for the investigation being reported.	*Scientific background and rationale are included, focusing on the prevalence of heightened state anxiety in the PED and the challenges that may result from it.*
3 Objectives	State specific objectives, including any prespecified hypotheses	*Purpose and objectives of the pilot study are stated, specifically that the purpose of this study was to gather preliminary data on the feasibility and effectiveness of utilizing audiobooks to reduce fear and state anxiety in children in kindergarten through 8th grade in the PED during a waiting gap in care.*
**Methods**		
4 Study design	Present key elements of study design early in the paper	*Study design (one-sample pre-posttest study) is stated in the first sentence of the Methods.*
5 Setting	Describe the setting, locations, and relevant dates, including periods of recruitment, exposure, follow-up, and data collection.	*Setting, location, and period of recruitment is described in Section 2; data collection details are described in Section 2.2 (Procedures).*
6 Participants	Give the eligibility criteria, and the sources and methods of selection of participants.	*Eligibility criteria was described in Section 2.1 (Participants); methods of selection of participants – a convenience sample of children presenting to the PED – is also described in Section 2.1.*
7 Variables	Clearly define all outcomes, exposures, predictors, potential confounders, and	*Outcome variables are clearly described in Section 2.3 (outcomes) and includes*

	effect modifiers.	*separate sections for Fear, State Anxiety, and Patient Experience.*
8 Data sources/ measurement	For each variable of interest, give sources of data and details of methods of assessment (measurement).	*Sources of data and details of methods of assessment for each variable of interest are described in Section 2.3 (outcomes).*
9 Bias	Describe any efforts to address potential sources of bias.	*Our IRB-recommended and approved assent language included the statement "We want to see if listening to an audiobook can make this time easier for children like you." It is possible that the language used in these assent procedures led to a priming effect, with children experiencing some anticipatory stress relief upon knowing they would be provided an audiobook to improve their experience in the PED. This is discussed in Section 4 (Discussion). Additionally, the use of a convenience sample is discussed as a limitation of the study in Section 4.3 (Limitations).*
10 Study size	Explain how the study size was arrived at.	*As the primary purpose of this study was to assess the feasibility of our intervention, we surmised a priori that a convenience sample of at least 100 participants would generate enough data achieve our primary aim.*
11 Quantitative variables	Explain how quantitative variables were handled in the analyses.	*Each quantitative variable was analyzed as-is. No data transformations were performed.*
12 Statistical methods	Describe all statistical methods. Explain how missing data were addressed.	*All statistical methods were described in Section 2.4 (Data Analysis); these included:*<br><br>*summary statistics (e.g., frequencies, means, standard deviations) to examine sample characteristics and participant experience, and chi-square tests to examine whether the patients who enrolled differed from those who declined, based on PED triage level and school grade. Paired samples t-test was used to examine pre-post intervention changes in fear and state anxiety. Pre-post differences in fear and state anxiety were computed for each individual participant and examined in the following ancillary analyses: Pearson's correlation test with child age and school grade level; one-way analysis of variance models with mental health status (yes or no), ED triage level*

		(urgent or emergent), and final diagnosis (six categories) as separate between-subjects variables. All analyses were conservatively two-tailed and conducted at the 0.05 significance level.<br><br>*Missing data: Prior to performing statistical analyses, we made the decision to exclude incomplete cases. As shown in Figure 1 and stated in the manuscript text: In order to examine the effectiveness of the full exposure of the audiobook intervention, discontinuations (e.g., lost interest, fell asleep) and/or withdrawn participants were excluded from analyses.*
**Results**		
13 Participants	Report all numbers of individuals at each state of study. Give reasons for non-participation at each stage. Consider use of a flow diagram.	*All numbers of individuals at each stage of the study were included in Figure 1 (study flow diagram). We did not formally ask participants their reason for non-participation, so have only limited information. We have data spontaneously provided by participants for 33% of individuals who declined participation; we can include this if Editors believe it would be beneficial.*
14 Descriptive data	Give characteristics of study participants. Indicate number of participants with missing data for each variable of interest.	*Characteristics of study participants is provided in Table 1 (characteristics of participants). As shown in Table 2, only one variable had missing data.*
15 Outcome data	Report numbers of outcome events or summary measures.	*All outcome data is reported in Section 3.2.1, 3.2.3, and/or Table 2.*
16 Main results	Give unadjusted estimates; report category boundaries when continuous variables were categorized.	*All outcome data is reported in Section 3.2.1, 3.2.3, and/or Table 2. Visual representation of data is presented in Supplementary Figures 2-4.*
17 Other analyses	Report other analyses done.	*Ancillary Results are reported in Section 3.2.2.*
**Discussion**		
18 Key results	Summarize key results with reference to study objectives.	*Key results are summarized.*
19	Discuss limitations of the study, taking	*Limitations are discussed in Section 4.3.*

Limitations	into account sources of potential bias or imprecision.	
20 Interpretation	Give a cautious overall interpretation of results considering objectives, limitations, multiplicity of analyses, results from similar studies, and other relevant evidence.	*Overall interpretation of results is provided in Section 4.4 (Conclusion).*
21 Generalizability	Discuss the generalizability (external validity) of the study results.	*Limitations to the generalizability of the study results are presented in the Limitations section (4.3) – specifically discussing limitations in study sample (only English-speaking participants), and use of a de novo measurement tool to assess anxiety.*
**Other Information**		
22 Funding	Give the source of funding and the role of the funders for the present study.	*Funding information is provided in Section 8 as well as Section 6 (Conflict of Interest), stating that Penguin Random House was not involved in the study design, collection, analysis, data interpretation, or dissemination of study results.*
